# Induction of peroxisome proliferator activated receptor γ (PPARγ) mediated gene expression and inhibition of induced nitric oxide production by *Maerua subcordata* (Gilg) DeWolf

**DOI:** 10.1186/s12906-020-2856-2

**Published:** 2020-03-12

**Authors:** Mebrahtom Gebrelibanos Hiben, Laura de Haan, Bert Spenkelink, Sebastiaan Wesseling, Jacques Vervoort, Ivonne M. C. M. Rietjens

**Affiliations:** 1 Division of Toxicology, Wageningen University & Research, Stippeneng 4, 6708 WE Wageningen, The Netherlands; 2 Department of Pharmacognosy, School of Pharmacy, College of Health Sciences, Mekelle University, 231 Mekelle, Ethiopia; 3 Laboratory of Biochemistry, Wageningen University & Research, Stippeneng 4, 6708 WE Wageningen, The Netherlands

**Keywords:** Peroxisome proliferator activated receptor gamma (PPARγ), Extracts, Gene expression, *Maerua subcordata*, Nitric oxide

## Abstract

**Background:**

The health benefits of botanicals is linked to their phytochemicals that often exert pleiotropic effects via targeting multiple molecular signaling pathways such as the peroxisome proliferator-activated receptors (PPARs) and the nuclear factor kappaB (NFκB). The PPARs are transcription factors that control metabolic homeostasis and inflammation while the NF-κB is a master regulator of inflammatory genes such as the inducible nitric-oxide synthase that result in nitric oxide (NO) overproduction.

**Methods:**

Extracts of *Maerua subcordata* (MS) and selected candidate constituents thereof, identified by liquid chromatography coupled to mass spectroscopy, were tested for their ability to induce PPARγ mediated gene expression in U2OS-PPARγ cells using luciferase reporter gene assay and also for their ability to inhibit lipopolysaccharide (LPS) induced NO production in RAW264.7 macrophages. While measuring the effect of test samples on PPARγ mediated gene expression, a counter assay that used U2OS-Cytotox cells was performed to monitor cytotoxicity or any non-specific changes in luciferase activity.

**Results:**

The results revealed that the fruit, root, and seed extracts were non-cytotoxic up to a concentration of 30 g dry weight per litre (gDW/L) and induced PPARγ mediated gene expression but the leaf extract showed some cytotoxicity and exhibited minimal induction. Instead, all extracts showed concentration (1–15 gDW/L) dependent inhibition of LPS induced NO production. The root extract showed weaker inhibition. Among the candidate constituents, agmatine, stachydrine, trigonelline, indole-3-carboxyaldehyde, plus ethyl-, isobutyl-, isopropyl, and methyl-isothiocyanates showed similar inhibition, and most showed increased inhibition with increasing concentration (1–100 μM) although to a lesser potency than the positive control, aminoguanidine.

**Conclusion:**

The present study demonstrated for the first time the induction of PPARγ mediated gene expression by MS fruit, root, and seed extracts and the inhibition of LPS induced NO production by MS fruit, leaf, root, and seed extracts and some candidate constituents thereof.

## Background

The well-known disease preventive and therapeutic potential of herbal medicines and foods rich in fruits, vegetables, and unrefined grains has been linked to their phytochemical content that often produce a pleiotropic physiological effects, targeting almost every molecular signaling pathway [1–3]. Among the vital pathways modulated by phytochemicals are the peroxisome proliferator-activated receptors (PPARs) and the nuclear factor kappaB (NF-κB) mediated signaling pathways.

Many dietary phytochemicals are PPAR activators and ligand binding to PPARs has been proven to control several pathological conditions linked with obesity, aging-related diseases, inflammation, immune disorder, cell cycle regulation as well as cancer [4]. The PPARs are a subfamily of ligand activated transcription factors that belong to the nuclear receptor superfamily. In mammals, three major PPAR isoforms (α, β /δ, and γ) control the expression of diverse genes involved in metabolic homeostasis, adipogenesis, and inflammation [5–9]. PPAR dependent regulation of transcriptional activity is mediated by PPAR:retinoid X receptor (RXR) heterodimers [10]. Upon ligand activation, the PPAR/RXR heterodimers, which are bound to specific DNA sequence elements termed peroxisome proliferator response elements (PPREs) in the regulatory region of their target genes, recruit specific cofactors, thereby stimulating the transcription of target genes [10–12]. PPAR ligand binding leads to interactions with co-activators and/or co-repressors to induce or inhibit their functions. The function of PPARs is mainly regulated through ligand binding but also by some post-translational modifications such as phosphorylation, SUMOylation, ubiquitination, and acetylation which are found at numerous modification sites [13].

PPARs control lipid metabolism and inflammation [10, 14]. Particularly, PPARγ activation is linked with beneficial health effects, including insulin-sensitization and immunomodulation with anti-inflammatory properties [12, 15]. While all three PPAR isotypes demonstrate anti-inflammatory effects, PPARγ was the first for which the mechanism by which it inhibits inflammation was elucidated [10, 16]. PPARγ undergoes ligand-dependent SUMOylation that results in its recruitment to the promoters of inflammatory genes where it inhibits transcription by stabilizing corepressor complexes. By this mechanism, PPARγ was shown to inhibit gene expression, in macrophages, of the NF-κB mediated inflammatory genes including the inducible nitric-oxide synthase (iNOS) gene [16, 17]. Likewise, PPARγ agonists attenuate the induction of iNOS expression by lipopolysaccharide (LPS) [17].

The NF-κB family of transcription factors are master regulators of immune and inflammatory processes [18–20] that induce the expression of various pro-inflammatory genes, including those encoding cytokines and chemokines [9]. For example, cytokines and gram negative bacterial endotoxins such as LPS were shown to cause NF-κB mediated induction of inducible nitric-oxide synthase (iNOS) gene expression and increased production of nitric oxide (NO) [21, 22]. Activating PPARγ gene expression or inhibiting NF-κB pathways likely has a protective effect against inflammatory diseases [23] and PPARγ agonists were shown to demonstrate anti-inflammatory effects [24] and inhibition of iNOS [25] by interfering with the NF-kB signalling pathways [26].

*Maerua subcordata* (Gilg) DeWolf (Capparidaceae/Capparaceae) is a medicinal and (famine) food plant. Its root tuber and leaf parts are used in traditional medicine to treat infections and for wound healing. Moreover, the root tuber is used to treat diabetes, high blood pressure, and allergic disorders as well as to improve appetite [27]. Inflammation is a common contributor to the pathology of all these disease conditions. It is now widely appreciated that low-grade chronic inflammation plays a key role in the initiation, propagation, and development of metabolic diseases, mainly in relation to obesity and type 2 diabetes, the metabolic syndrome, cancer, and cardiovascular diseases [18, 28, 29]. Although cure of inflammatory diseases is a significant challenge, medicinal herbs used in traditional medicine may signify a possible option for obtaining effective anti-inflammatory therapies [30]. Especially, Brassica vegetables are known for their preventive role against these inflammation related disorders, mainly due to their glucosinolate content. Glucosinolate hydrolysis products, the isothiocyanates, are known to play important roles in disease prevention by triggering antioxidant and anti-inflammatory responses, among others [31–34]. In our previous work, the nuclear factor (erythroid-derived 2)-like 2 (Nrf2) mediated antioxidant effect of *M. subcordata* different extracts and the presence, in these extracts, of phytochemicals such as glucosinolates was reported [35]. Thus, taking the above viewpoints into account, the present study is aimed to identify further endpoints that signify health benefits by assessing the potential of *M. subcordata* extracts and selected candidate constituents thereof, for induction of PPARγ mediated gene expression and inhibition of NF-kB/iNOS mediated NO production in LPS-activated RAW264.7 macrophages.

## Methods

### Chemicals and reagents

Arecaidine hydrochloride was from Alfa Aesar (Karlsruhe, Germany); N-acetylagmatine from Cayman Chemicals-Europe (Sanbio Uden, The Netherlands); glucobrassicin potassium salt, stachydrine hydrochloride, and trigonelline hydrochloride from PhytoLab (Vestenbergsgreuth, Germany); agmatine sulfate, anthranilic acid, azeleic acid, bovine serum albumen (BSA), dimethyl sulfoxide (DMSO), 3-(4,5-dimethylthiazolyl)-2,5-diphenyltetrazolium bromide (MTT), ethanol, indole-3-carboxaldehyde, isothiocyanates (methyl-, ethyl-, isobutyl-, isopropyl-isothiocyanates), geranylgeranylacetone, 9-hydroxyoctadecadienoic acid, α-linolenic, α-lipoic acid, lipopolysaccharide (from *Escherichia coli* 055:B5-γ-irradiated, BioXtra, suitable for cell culture), N-1-napthylethylenediamine dihydrochloride (NED), petroselinic acid, pipecolic acid, rosiglitazone, sclareol, stigmasterol, RRR-α-tocopherol, and Viscozyme L were from Sigma-Aldrich (Germany and The Netherlands), sulfanilamide (Sigma, China); ortho-phosphoric acid 85% and sodium nitrite were from Merck (Darmstadt, Germany). Minimum Essential Medium alpha 1:1 mixture of Dulbecco’s modified Eagle’s medium and Ham’s F12 medium (DMEM/F12)(with and without phenol red), foetal calf serum (FCS), and Phosphate Buffered Saline (PBS) were from Gibco life technology (Paisley, UK); trypsin, nonessential amino acids (NEAA), and G418 were from Invitrogen Corporation (Breda, The Netherlands); and dextran-coated charcoal-stripped foetal calf serum (DCC-FCS) was purchased from Thermo Scientific (Waltham, USA).

### Plant material

Different parts of *M. subcordata* (fruit, leaf, root tuber, and seed) were collected near Shiraro area (14.3970° N, 37.7743° E) of Northwest Tigray, Northern Ethiopia. *M. subcordata* is a wild shrub, sometimes considered as invasive weed, which does not raise concerns of endangered or protected species. Collection of plant material was made from unreserved and publically open area where the local traditional healers also collect their target medicinal plants and hence no special permission was required to make the collection. Plant authentication was performed by Mr. Melaku Wendafrash in the National Herbarium at Addis Ababa University, Addis Ababa, Ethiopia where a specimen (Voucher number MG001/2007) was deposited. The plant parts were sorted and dried in the laboratory of Pharmacognosy, Mekelle University, Mekelle Ethiopia. The root tuber was sliced into small pieces after its outer thin coating was peeled and the sliced pieces dried in an oven at a temperature of 40 °C for four days. The other parts (fruit, leaf, and seed) were dried in the shade at room temperature and seeds were taken out of fruits after drying. The dried plant materials were packed in plastic bags, and stored at room temperature on shelf until they were transported to Wageningen University, The Netherlands; where they were powdered: each dried plant part was splashed with liquid nitrogen to remove moisture and facilitate powdering, and then powdered using an analytical miller. Each powdered plant material was mixed well, packed in capped plastic tubes, and stored at − 80 °C until further use.

### Cell lines

Cytotox CALUX and PPARγ2 CALUX cells (Bio Detection Systems, Amsterdam, The Netherlands) as well as RAW264.7 murine macrophage-like cells (American Type Culture Collection) were used in the present study. The cytotox CALUX cells are human osteosarcoma U2OS cells stably transfected with a reporter construct carrying a luciferase reporter gene under transcriptional control of a constitutive promoter. They have an invariant luciferase expression and serve to determine cytotoxicity and to investigate whether stabilisation of the luciferase enzyme is occurring during chemical exposure [36, 37]. The PPARγ2 CALUX cells are human osteosarcoma U2OS cells stably transfected with an expression vector for PPARγ2 and a reporter construct containing a luciferase gene under transcriptional control of the peroxisome proliferator responsive element [38, 39]. Both PPARγ2 and Cytotox CALUX cells were cultured in DMEM/F12 glutamax supplemented with 7.5% FCS, and 1% NEAA. Once per week 200 μg/mL G418 was added to the culture medium in order to maintain selection pressure [38]. RAW264.7 cells are from a macrophage-like cell line derived from Balb/c mice. They maintain many of the properties of macrophages including nitric oxide production [40]. The RAW264.7 cells were grown and maintained in DMEM/F12 glutamax supplemented with 10% FCS, and 1% NEAA [41]. All cell lines were incubated at 37 °C and 5% CO_2_.

### Preparation of extracts

Enzyme (Viscozyme L) hydrolysed and non-hydrolysed methanol extracts from dried powders of *M. subcordata* fruit, leaf, root tuber, and seed samples were prepared following the procedure described by Gijsbers et al. [36] and Hiben et al. [35]. To prepare non-hydrolysed extracts, 3.4 ml methanol was added to 0.6 g plant material and vortexed. The mixture was sonicated (10 min), centrifuged (15 min, 1000 g), the supernatant of each sample filtered using 0.2 μm polytetrafluoroethylene (PTFE)-filters (Whatman, Germany), and freeze-dried after the methanol was evaporated under a stream of nitrogen. Dried extracts were stored at − 80 °C until further use. These extracts were re-dissolved in DMSO:DMEM (without phenol red)(1:2 v/v) [36] when preparing exposure medium for the assays with CALUX cell lines and in DMEM when preparing exposure medium for the assays with RAW264.7 cells. To prepare enzyme hydrolysed extracts, 300 μl sodium acetate (0.1 M, pH 4.8) and 100 μl of Viscozyme L were added to 0.6 g plant material, followed by 1 h incubation in a water bath at 37 °C. Then, samples were put on ice and 3.0 ml methanol was added to each sample, followed by 10 min sonication and 15 min centrifugation at 1000 g. The supernatant was then filtered, freeze-dried and stored at − 80 °C until further use similar to the procedure for the non-hydrolysed extracts. These enzyme-hydrolysed extracts were re-dissolved in DMSO: DMEM (without phenol red) (1:4 v/v) [36] while preparing exposure medium for the assays with CALUX cell lines.

### CALUX assays

The PPARγ mediated gene expression induction potential of *M. subcordata* methanol extracts and selected candidate constituents was assessed by measuring induction of luciferase activity in PPARγ2 CALUX luciferase reporter cells. For each assay with PPARγ2 CALUX cells, an identical counter screen was done using the U2OS cytotox CALUX cells. Each plate included rosiglitazone (1 μM or 10 μM) as positive control and 1% (v/v) DMSO as a solvent control. In each well containing test samples, the final DMSO concentration was 1% (v/v). Assays were performed essentially as described by Gijsbers et al. [38] and Beekmann *et al*. [39]. Briefly, the CALUX cells were seeded in the 60 inner wells of a white 96-well view plate at a density of 1 × 10^4^ cells per well in 100 μl assay medium consisting of DMEM/F12 without phenol red supplemented with 5% DCC-FCS, and 1% NEAA. The outer 36 wells were filled with 200 μl PBS to maintain physical homogeneity throughout the plate. While screening fatty acids, cells were seeded with assay medium containing 50 μM vitamin E (prepared by adding 20 μL of a 50 mM RRR-α-tocopherol solution to 20 mL of assay medium). The seeded cells were incubated for 24 h to allow them to attach and form a confluent monolayer. The next day, 100 μl exposure medium containing the test samples was added to each well resulting in 200 μl assay medium per well. An assay medium containing 50 μM freshly added vitamin E and 0.1% BSA was used when preparing exposure medium containing fatty acids, and both positive and solvent controls. The vitamin E serves as an antioxidant to prevent oxidation of the unsaturated fatty acids while BSA facilitates the solubility and cellular availability of the fatty acids. After 24 h exposure, medium was removed, cells were washed with ½ PBS (PBS half diluted with nano pure water) and then 30 μl low salt buffer was added to each well with cells. The plates were subsequently frozen overnight at − 80 °C in order to lyse the cells. Then plates were thawed and luciferase activity per well in the lysate was measured in relative light units (RLU) using a luminometer (GloMax-Multi Detection System-Promega, USA) upon adding 100 μl per well of flash mix. Results were described as fold induction compared to the solvent control. Extracts or compounds giving less than twofold induction at the highest concentration that could be tested without cytotoxicity were considered unable to induce PPARγ - mediated luciferase gene expression.

#### Alamarbleu (resazurin) assay

In addition to the cytotox reporter gene assay, the cytotoxicity of test samples was evaluated by the Alamarbleu (resazurin) assay. Cytotox CALUX and PPARγ2 CALUX cells were cultured in 96 well plates in their appropriate medium, described above, for 24 h and then the cells were exposed to test samples for another 24 h. Then alamarbleu (resazurin) reagent solution (10% w/v in PBS) was added directly to the cells (10% v/v i.e. 20 μl reagent to 200 μl cells in medium). Following reagent addition, plates were covered by aluminium foil, incubated for 2 h after which fluorescence was measured (λex 570/λem 585) using a plate reader (Molecular Devices, Spectra Max M2) equipped with Softmax Pro software.

### Inhibition of nitric oxide production in RAW264.7 macrophages

The inducible nitric oxide synthase (iNOS) mediated nitric oxide production inhibition capacity of *M. subcordata* extracts and selected constituents was assessed using gram negative bacterial lipopolysaccharides (LPS)-stimulated RAW264.7 mouse macrophages. Assays were carried out essentially as described by Meijerink *et al*. [41]. Briefly, adherent RAW264.7 macrophage cells were scraped, suspended in fresh medium, diluted to a final density of 5 × 10^5^ cells/ml, and seeded by pipetting 100 μl (5 × 10^4^ cells/well) of the cell suspension to the inner 60 wells of 96 well plates (transparent flat bottom). The outer wells were filled with 200 μl PBS and the plates were incubated for 24 h. Stock solutions of each extract (in DMEM) and each candidate compound (in DMSO) were used to prepare exposure medium by serial dilution using medium alone and medium with LPS (400 ng/ml), each test concentration diluted 1000x. After careful aspiration of the culture medium, cells were exposed to the test samples by pipetting 100 μl of exposure medium containing each test concentration in triplicate. The DMSO final concentration was set to be 0.1% (v/v). Two solvent controls (medium only and medium with 0.1% (v/v) DMSO) were used, both with and without LPS. After 24 h exposure, the supernatant medium in each well was transferred into a new plate to measure the nitrite level using the Griess assay and 100 μl fresh medium were added to the remaining cells which were used for the MTT assay to assess cell viability.

#### The Griess assay

*Assay principle*: in biological systems, NO is rapidly oxidized by oxygen to nitrite (NO_2_^−^) and/or nitrate (NO_3_^−^), its two primary, stable and non-volatile breakdown products. The measurement of nitrate/nitrite concentration is routinely used as an index of NO production [42]. Measuring nitrite concentration, which relies on a diazotization reaction that was originally described by Griess in 1879, is a reliable method for quantifying NO production by cells [43]. Nitrite is first treated with a diazotizing reagent, sulfanilamide (SA), in acidic media to form a transient diazonium salt which is then allowed to react with a coupling reagent, N-1-naphthyl-ethylenediamine (NED), to form a stable azo compound. The intense purple colour of the product allows nitrite quantification at concentrations as low as ~ 0.5 μM. The absorbance of this adduct at 540 nm is linearly proportional to the nitrite concentration in the sample [42, 44]. The present study followed this principle to measure the nitrite level, and thereby effects of test samples on NO production by cultured RAW264.7 macrophages*.* To this end, the supernatant of exposed cells in each well was transferred into a new plate. In empty rows of wells of the same plate, different concentrations (0 to 100 μM) of standard sodium nitrite were prepared in duplicate. Then 50 μl of 1%(w/v) SA in 5% phosphoric acid was added to all wells, and after 10 min incubation at room temperature, 50 μl 0.1%(w/v) NED dihydrochloride was added to all wells. Absorbance was measured at 540 nm using a plate reader (Molecular Devices, Spectra Max M2) within 10 to 30 min after reagent addition. NO production was estimated from the absorbance reading using an equation from a standard nitrite calibration curve. To show that induction of NO production by LPS was evident, exposures with and without LPS (LPS^+^ and LPS^−^, respectively) were related in each plate for each test concentration by comparing NO values of each test exposure (NO [t]) to NO value from LPS^+^ medium control, representing maximum LPS-induced NO production (NO [M]). As the value of NO [M] may vary for different tests, and also to present results from different tests in one graph, data were further harmonized by dividing the mean NO [t] by the mean NO [M] value so that the NO [M] value for all tests represent 100%. %NO production = (mean NO [t]/mean NO [M])× 100. For exposures with LPS, the %NO production values below 100% indicate inhibition of LPS induced NO production or otherwise cytotoxicity whereas values above 100%, if any, may indicate enhancement of LPS induced NO production by test samples. With the assumption that NO value from exposures to LPS^−^ medium control should represent background absorbance (0% LPS-induced NO production) while NO value from exposures to LPS^+^ medium control represent maximum measurement (100% LPS-induced NO production), percent inhibition of LPS-induced NO production by test samples was calculated from corrected NO values (background absorbance subtracted).

%Inhibition = {(meanNO [Mc] - mean NO [tc])/meanNO [M]}× 100, where mean NO [Mc] is the mean value of corrected NO production from exposure to medium plus LPS and mean NO [tc]) is the mean value of corrected NO production from an exposure to each test concentration.

#### MTT assay

The initial plates with the remaining cells were used in this assay. MTT reagent solution (5 mg/ml in PBS) was added directly to the cells (10% v/v i.e. 10 μl reagent/100 μl medium). Following reagent addition, plates were covered by aluminium foil, incubated for 1 h, after that medium was carefully aspirated and formazan crystals were dissolved in 100% DMSO after which absorbance was measured at 570 nm using a plate reader (Molecular Devices, Spectra Max M2) equipped with Softmax Pro software.

### Metabolic profiling of *M. subcordata* extracts by LC-MS/MS

The identification of constituents in the methanol extracts of *M. subcordata* (fruit, leaf, root, and seed) was achieved employing liquid chromatography (LC) coupled with multistage mass spectroscopy (MS) as described previously [35]. Briefly, methanol extracts of different plant parts were subjected to LC-MS analysis and generated spectral data were uploaded to ‘Ms Annotation based on in silico Generated Metabolites’ (MAGMa) for structural annotation. MAGMa is an application software (http://www.emetabolomics.org/magma) offered by Ridder et al. [45] to help structural characterization of mixtures of metabolites present in complex extract samples. The MAGMa user interface displays alternative candidate structures, retrieved from Kegg, that are ranked on the basis of calculated matching score. The matching score helps to select candidate structures, from the user interface display, that are most probable constituents of the sample extracts. The identity of selected candidates was further confirmed and quantified by LC-MS/MS based on multiple reaction monitoring (MRM) and standard calibration curves. Optimum LC-MS/MS operating conditions were set using commercially available standard compounds. Varying concentrations of standard solutions, used to generate calibration curves, and filtered (0.2 μm polytetrafluoroethylene (PTFE)-filter) extract samples were analysed under identical conditions. The compounds of interest in the sample extracts were identified by their retention time and quantified based on calibration curves.

### Data analysis

For each experiment, at least three independent tests were performed. Graphs present the average of repeated tests. Data were analysed using Microsoft excel 2016. For the CALUX assays, data are expressed as fold induction over the solvent control, and presented as mean values ± standard deviation (SD). For assays with RAW264.7 macrophages, data are described as percent nitric oxide (NO) production or as percent inhibition of LPS induced NO production after subtracting the background absorbance from treatments without LPS. Each data point was measured, at least, in triplicate. Statistical significance was assessed using SPSS 23, paired samples statistics t-test. Moreover, the results from assays with RAW264.7 macrophages were compared by one-way analysis of variance (ANOVA) followed by Tukey HSD Post Hoc tests.

*P* ≤ 0.05 was considered statistically significant.

## Results

### Effect of *M. subcordata* methanol extracts on viability of U2OS CALUX cells

**Fig. 1:**
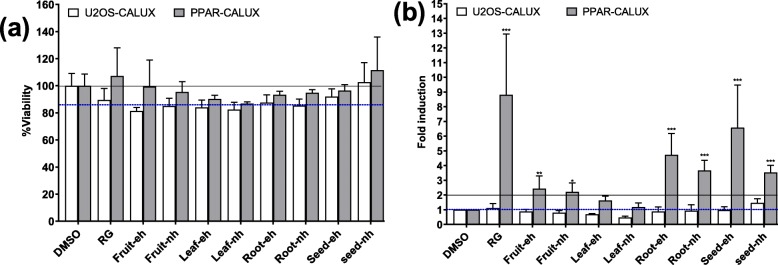
Effect of *M. subcordata* extracts on viability and luciferase activity of U2OS-Cytotox and U2OS-PPARγ cells. Showing the effect after 24 h exposure to rosiglitazone (RG,10 μM), enzyme hydrolysed (eh) and non-hydrolysed (nh) methanol extracts of fruit, leaf, root and seed of *M. subcordata* tested at a concentration of 30 gDW/L. Cell viability (**a**) was measured by the resazurin assay with the viability of cells exposed to the solvent control (1% DMSO) set at 100%, and luciferase activity **(b**) was measured by the CALUX reporter gene assay and expressed as fold induction compared to solvent control. Data are presented as mean ± SD` of three independent experiments. Asterisks indicate a significant difference from the solvent control: *p < 0.05; **p < 0.01; ***p < 0.001

Results of the resazurin assay (Fig. 1a) show that all the tested extracts up to a concentration of 30 gDW/L (gram dry weight per litre) were not cytotoxic (cell viability ≥80%) [46] to both U2OS-Cytotox CALUX and U2OS-PPARγ CALUX cell lines. 30 gDW/L was used as screening concentration for the extracts in further reporter gene assays.

### Induction of PPARγ mediated luciferase expression by *M. subcordata* extracts

Results of the U2OS-PPARγ CALUX assay (Fig. 1b) show that both enzyme hydrolysed (eh) and non-hydrolysed (nh) extracts from the fruit, root, and seed materials as well as rosiglitazone (positive control) increased luciferase activity compared to 1% (v/v) DMSO as a solvent control implying induction of PPARγ mediated gene expression, whereas the induction by the leaf extracts was minimal. For all plant parts, the enzyme hydrolysed extracts showed slightly higher increase in luciferase activity than the matching non-enzyme hydrolysed extracts. Generally, the seed extract showed the highest induction of PPARγ mediated gene expression followed by the root extracts and then the fruit extracts though the difference was not statistically significant. To monitor non-specific interference with luciferase activity or cytotoxicity by the extracts, a parallel test was done using the U2OS-cytotox assay. Unlike the resazurin assay that showed non-cytotoxic effects for all the tested extracts, the cytotox assay showed that the leaf extracts reduced luciferase activity (69 and 47% remaining luciferase activity compared to the solvent control for eh and nh extracts, respectively) implying that the leaf extracts may have caused cytotoxicity or may have interfered with luciferase activity, ultimately resulting in minimal increase in luciferase activity. On the other hand, the fruit, root, and seed extracts showed similar activity as the solvent control indicating the absence of cytotoxicity in line with the resazurin assay. As luciferase activity at 30 gDW/L in the U2OS-cytotox assays were similar to that of the solvent control for these extracts, it was assumed that the extracts were non-cytotoxic and did not interfere with luciferase itself. Thus, induction of PPARγ mediated gene expression by the fruit, root, and seed extracts observed in the U2OS-PPARγ CALUX assay was not caused by stabilization of the luciferase but reflected induction of gene expression.

### Some selected candidate compounds in *M. subcordata*

**Fig. 2:**
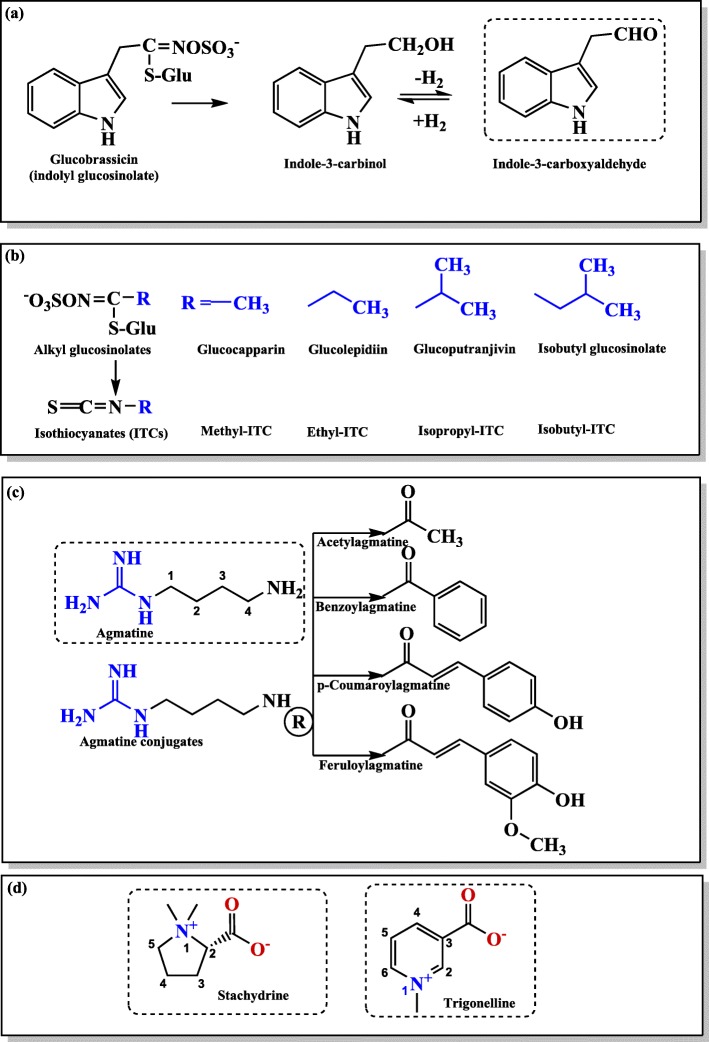
Representative phytochemical groups and constituents identified in *M. subcordata*. Boxes **a** and **b** show glucosinolates and their break down products, box **c** presents guanidines, and box **d** quaternary amines. Dotted boxes show candidates the presence of which was confirmed using standards

In an attempt to identify phytochemicals of *M. subcordata* with possible effect on the induction of PPARγ mediated gene expression and/or inhibition of inflammation pathways, LC-MS/MS analysis plus MAGMa software based structural annotation was done on the methanol extract of each plant part that resulted in the tentative identification of different constituents from which candidates were selected based on literature reports (S1 Table). Whereas the identification of constituents such as glucosinolates and some biogenic amines was reported in our previous work [35], the present report provides additional candidate constituents including guanidine derivatives (S1a Fig), quaternary ammonium compounds (betaines) (S1b Fig), and fatty acids and miscellaneous compounds (S1c Fig). Further LC-MS analysis using reference standard compounds confirmed the presence of agmatine in all extracts and of indole-3-carboxyaldehyde (I3C) in fruit, leaf, and seed extracts of *M. subcordata* (Table 1). I3C is a breakdown product of glucobrassicin (GluB) (Fig. 2a), an indolyl glucosinolate previously reported in *M. subcordata* with some aliphatic glucosinolates, stachydrine, and trigonelline [35]. The detection of I3C only in fruit, leaf, and seed extracts supports our previous report that GluB was detected only in these extracts. Besides, fatty acids with reported PPARγ agonist activity were part of the tentative identification but their definite identification was not accomplished in the present report and needs further work. Some selected candidates that were tested for their potential effect on induction of PPARγ mediated gene expression and/or inhibition of NO production in further experiments are presented in Fig. 2a-d.

**Table 1:** Agmatine and indole-3-carboxyaldehyde in *M. subcordata* extracts. Identified and quantified by LC-MS/MS-MRM and standard calibration curves: (**a**) amount described in microgram per gram dry weight (μg/gDW) and (**b**) extrapolated concentration (μM) in 96 well plate as calculated from the maximum concentration (30 gDW/L) which was applied in the in vitro studies with plant extracts. Rt, retention time

Samples	Compounds			
	Agmatine; Rt = 0.81 min		Indole-3-carboxyaldehyde; Rt = 9.51 min	
	a	b	a	b
Fruit	66.65	15.36	1.32	0.27
Leaf	24.51	5.65	0.84	0.17
Root	13.84	3.19	0.00	0.00
Seed	42.37	9.76	12.63	2.61

### Induction of PPARγ mediated luciferase expression by candidate constituents of *M. subcordata*

Different candidate constituents were screened for their PPARγ-mediated luciferase expression potential. The results (Table 2) revealed that α-lipoic acid and some candidate fatty acids such as α-linolenic acid, 9-hydroxyoctadecadienoic acid (9-HODE), and petroselinic acid showed biologically relevant (≥2 fold) induction of luciferase activity while the induction by the other screened candidates was minimal.

**Table 2:** Selected candidate constituents in *M. subcordata* screened for their potential induction of PPARγ-mediated luciferase expression

Compounds	Concentration (μM)	Fold induction (mean ± SEM)
**(a)***Fold induction compared to 1%(v/v) DMSO as the solvent control*		
Agmatine sulfate	100	1.35 ± 0.09
N-Acetylagmatine	100	1.17 ± 0.30
Anthranilic acid	100	1.89 ± 0.33
Arecaidine hydrochloride	100	0.95 ± 0.03
Stachydrine hydrochloride	100	1.04 ± 0.19
Trigonelline hydrochloride	100	1.09 ± 0.11
Pipecolic acid	100	0.88 ± 0.02
Indole-3-carboxaldehyde	100	1.51 ± 0.11
Glucobrassicin potassium	10	1.05 ± 0.08
Ethyl isothiocyanate	10	1.18 ± 0.20
Isobutyl isothiocyanate	10	1.91 ± 0.62
Isopropyl isothiocyanate	10	1.76 ± 0.52
*sec*-Butyl isocyanate	10	1.27 ± 0.14
Geranylgeranylacetone	25^ϕ^	1.0 ± 60.06
Sclareol	5^ϕ^	1.15 ± 0.12
α-Lipoic acid	250	2.16 ± 0.27*
**(b)***Fold induction compared to 1%(v/v) ethanol as the solvent control*		
Rosiglitazone	1	15.09 ± 0.6**
9-Hydroxyoctadecadienoic acid	10^#^	2.13 ± 0.15*
α-Linolenic acid	100	2.93 ± 0.23*
Petroselinic acid	100	2.22 ± 0.53*
Azeleic acid	100	1.31 ± 0.08
Stigmasterol	100	1.49 ± 0.17

^ϕ^highest non-cytotoxic concentration, ^#^amount of sample was not enough to test higher concentration, *significant difference from the solvent control: *p < 0.05; **p < 0.01

### Inhibition of LPS induced nitric oxide production in RAW264.7 macrophages by *M. subcordata*

**Fig. 3:**
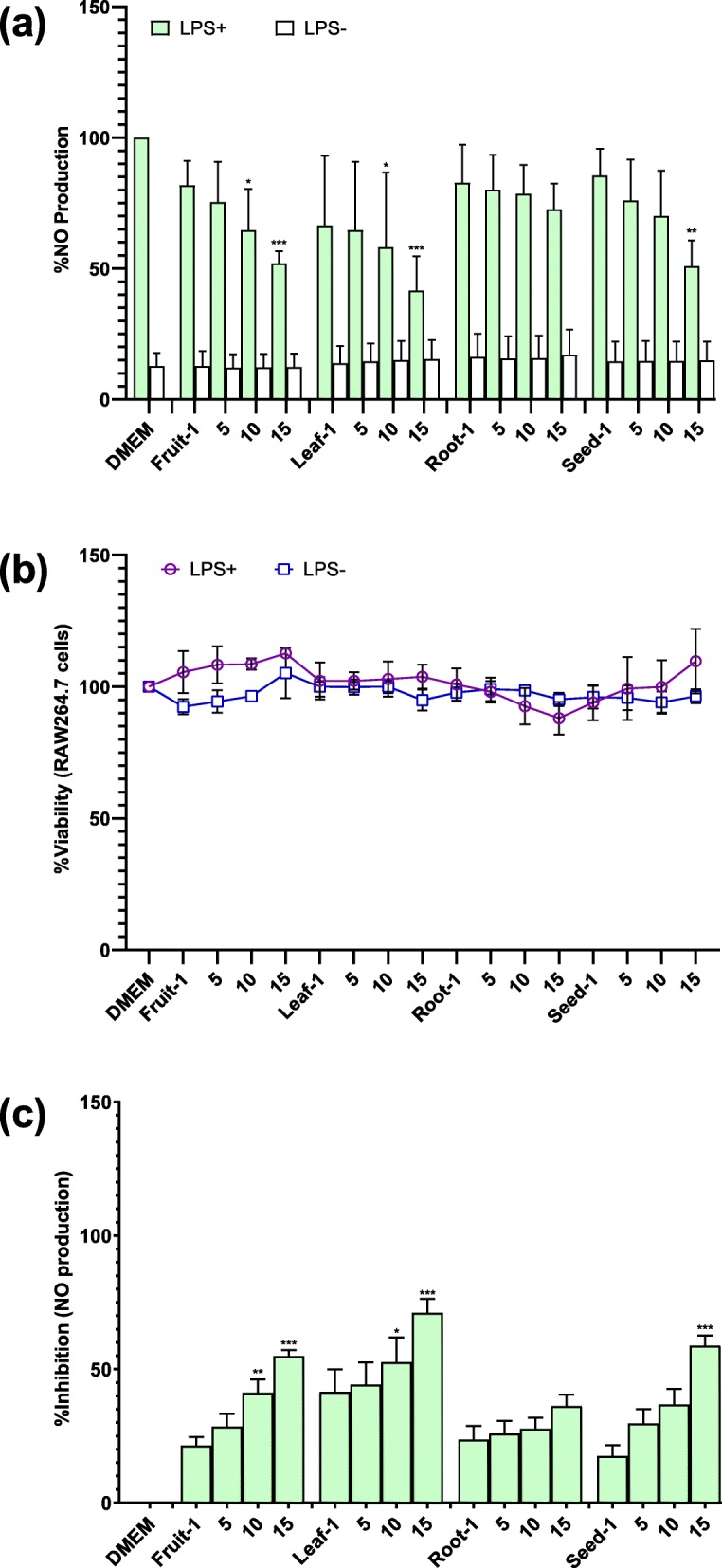
Effect of *M. subcordata* extracts on NO production in LPS-induced RAW264.7 macrophages. Showing the effect of the tested extracts, as compared to the medium control, on (**a**) NO production by cells with and without LPS (LPS^+^ and LPS^−^) treatments as measured by the *Griess assay*, **(b)** cell viability as measured by the *MTT assay*, and **(c)** description of %inhibition NO production. Data are presented as mean ± SD and asterisks show a significant difference from control (medium + LPS): *p < 0.05; **p < 0.01; ***p < 0.001

**Fig. 4:**
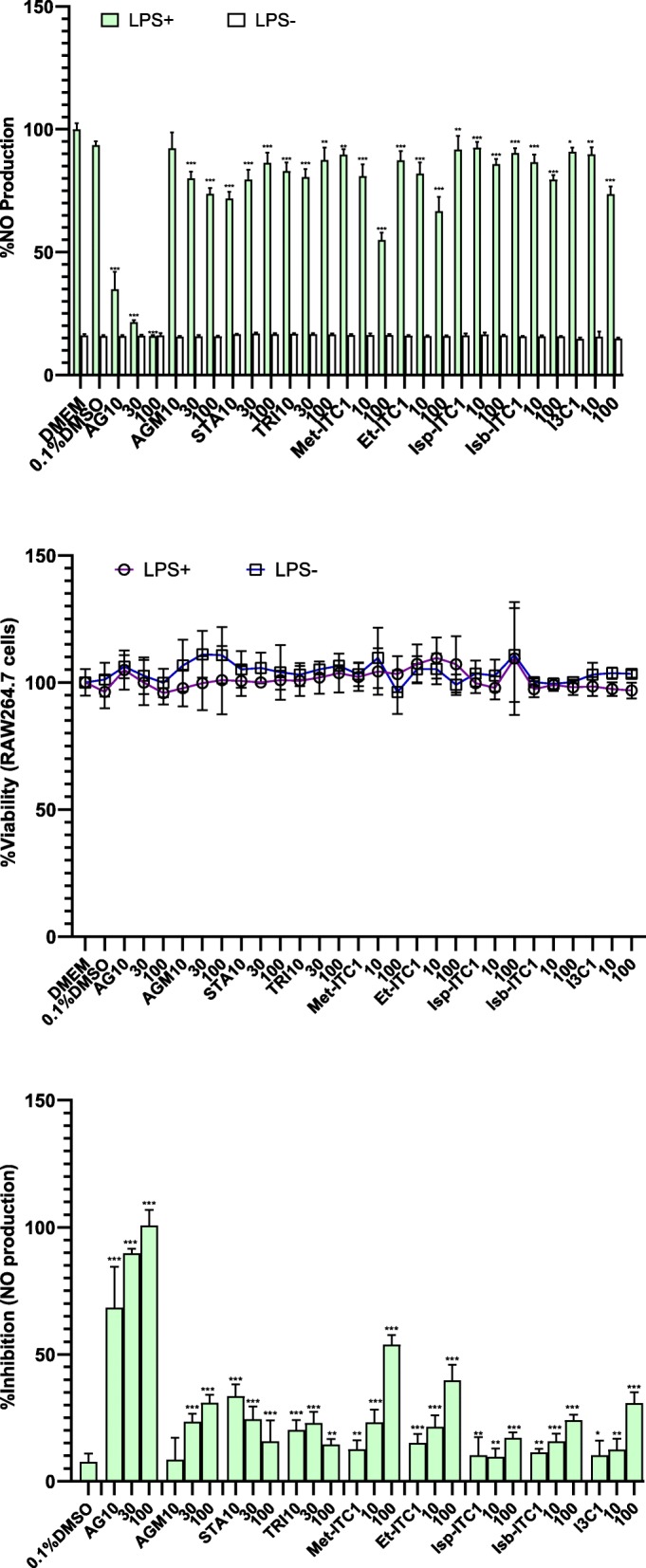
Effect of *M. subcordata* candidate constituents on NO production in LPS-induced RAW264.7 macrophages. Showing the effect of aminoguanidine (AG) as a positive control, the tested candidates including agmatine (AGM), stachydrine (STA), trigonelline (TRI), indole-3-carboxaldehyde (I3C), and the isothiocyanates (ITCs): methyl- (Met-ITC), ethyl- (Et-ITC), isopropyl- (Isp-ITC) as compared to medium control. (**a**) NO production by cells with and without LPS (LPS^+^ and LPS^−^) treatments as measured by the *Griess assay*, (**b)** cell viability as measured by the *MTT assay*, and (**c)** description of %inhibition NO production. Data are presented as mean ± SD and asterisks show a significant difference from control (0.1%DMSO + medium + LPS): *p < 0.05; **p < 0.01; ***p < 0.001

As a measure of a possible anti-inflammatory effect, the potential inhibition of LPS induced nitric oxide (NO) production in RAW264.7 macrophages by *M. subcordata* methanol extracts and selected candidate constituents identified in these extracts were evaluated. LPS induced NO production was estimated using an equation from a standard calibration curve (S2a Fig). To check for any possible influence of the extracts on absorbance at 540 nm or if NO radical scavenging activity by the extracts may contribute to any change in absorbance, a test with and without addition of extracts (10 and 50 gDW/L final concentrations) to the standard nitrite (S2b Fig) was done which revealed no noticeable intrinsic influence by the extracts. Figures 3a and 4a show results described as %NO production revealing that exposures without LPS (LPS^−^) reflect background levels and the background NO value for all test samples is comparable to that of the medium control. This implies the test samples had no influence on the absorbance reading emanating from intrinsic property or induction of cytotoxicity. To further show that reduction in NO production by the tested samples was not due to cytotoxicity, the cell viability MTT assay was performed, which showed that the extracts (Fig. 3b) as well as the tested compounds (Fig. 4b) were non-cytotoxic at the tested concentrations. Finally, to show the net inhibition (free of background values) of NO production by the test samples, results (Figs. 3c and 4c ) were described as percent inhibition of NO production. These results show that all *M. subcordata* (fruit, leaf, root, and seed) extracts revealed statistically significant (*p* < 0.05) inhibition of LPS induced NO production (Fig. 3c). Also, the tested candidate constituents and aminoguanidine, used as a positive control, inhibited LPS induced NO production (Fig. 4c). These results obtained (Fig. 4c) reveal that many of the screened candidates show inhibition of LPS induced NO production, most of them in a concentration dependent manner, albeit to a weaker extent than the positive control, aminoguanidine. While stachydrine tended to show a concentration dependent decline in inhibitory activity, the activity by trigonelline seems to be independent of concentration, if not slightly decreasing with increasing concentration.

## Discussion

The present study reports the induction of peroxisome proliferator activated receptor γ (PPARγ) mediated gene expression in U2OS-PPARγ cells and the inhibition of gram-negative bacterial lipopolysaccharide (LPS) induced nitric oxide (NO) production in RAW264.7 macrophages by *M. subcordata* extracts and candidate constituents thereof.

PPARs, a unique set of fatty acid regulated transcription factors that control lipid metabolism and inflammation [10, 14], have emerged as therapeutic targets to treat various components of the metabolic syndrome [10]. In this regard, botanicals of dietary or medicinal products have attracted more and more attention owing to their relative effectiveness in upregulating PPAR signalling with fewer significant side effects [47]. Accordingly, *M. subcordata* fruit, root, and seed parts may provide possible health benefits by this mechanism as extracts from these plant parts were shown to exhibit PPARγ mediated induction of luciferase gene expression.

Metabolic syndrome is a cluster of risk factors often associated with obesity and characterized by macrophage infiltration and activation in adipose tissue and liver [10]. The nuclear factor kappaB (NF-κB) mediated induction of inducible nitric oxide synthase (iNOS) gene expression is up-regulated in tissues affected by inflammatory responses and in macrophages in response to inflammatory stimuli or pro-inflammatory cytokines that result in overproduction of NO [21, 22, 43]. Although NO is an important molecule, involved in regulation of many physiological and micro biocide processes, its overproduction is implicated in the pathogenesis of several chronic inflammatory and immunologically mediated diseases, and in complications of diabetes and obesity [43, 48–50]. As NO is a key mediator of inflammation [48, 49], inhibition of inflammatory stimuli-induced NO accumulation has been suggested as a beneficial therapeutic strategy [51]. Natural products including medicinal plants provide a vast pool of NO inhibitors [52]. Likewise, extracts of *M. subcordata* (fruit, leaf, root, and seed) inhibited LPS-induced NO production in RAW264.7 macrophages to a varying extent, with the root extract showing weak inhibition. Since *M. subcordata* is used in traditional medicine to manage infectious and chronic diseases, and since inflammation is a common contributor to the pathology of these diseases, the inhibition of NO production by *M. subcordata* extracts may partly justify its traditional claims and also may show its potential as anti-inflammatory agent. Moreover, selected candidate constituents from this plant exhibited induction of PPARγ mediated gene expression or inhibition of NO production, which may substantiate its anti-inflammatory potential.

In line with the established evidence that endogenous or dietary fatty acids are known ligands of PPARs [53, 54], the candidate fatty acids α-linolenic acid, 9-hydroxyoctadecadienoic acid (9-HODE), and petroselinic acid showed biologically relevant (≥2 fold) [55] induction of luciferase activity in the present work (Table 2). On the other hand, although an in vivo study in rats showed that agmatine increased the gene expression (levels of mRNA) of PPARα and PPARγ [47], both agmatine and its acetyl conjugate did not induce an increase in luciferase expression at the protein level in the present study. This may happen since a gene’s mRNA level does not usually predict its protein level [56] due to unpredictable changes during translation. Stachydrine and trigonelline are constituents of medicinal plants used in traditional medicine for treatment of diabetes and the metabolic syndrome [57, 58]. Both compounds showed a PPARγ receptor glide score (ligand binding free energy) comparable to synthetic antidiabetic drugs [57]. Yet, no activity above solvent control was detected for stachydrine in luciferase reporter gene assays of all three PPAR (α, β/δ, and γ) isoforms [58] while reports on trigonelline seem contradicting. Trigonelline increased insulin sensitivity and enhanced adipose tissue PPARγ activity in diabetic rats in vivo [59]. However, in in vitro tests, trigonelline showed either no effect [60] or down regulated [61] PPARγ gene expression in cultured cells. In line with these reports, both compounds showed no induction of PPARγ mediated luciferase activity above the solvent control in the present study. Overall, most tested candidates, except for some fatty acids, showed no biologically relevant induction of PPARγ mediated-luciferase expression (Table 2) and hence were considered inactive.

Alternatively, most tested candidate constituents including agmatine, stachydrine, trigonelline and some break down products of glucosinolates like methyl-, ethyl-, isobutyl-, and isopropyl-isothiocyanates, and indole-3-carboxyaldyhyde were shown to inhibit NO production in LPS-stimulated RAW264.7 macrophages, albeit to an extent less potent than aminoguanidine, a selective iNOS inhibitor used as a positive control. The candidates that showed little or no PPAR*γ*-mediated luciferase activity, though reported as PPAR*γ* ligands and/or to impact PPAR*γ* functions, inhibited NO production, which may be justified by the fact that the mechanism by which PPARγ and its agonists inhibit iNOS expression and NO production requires ligand-dependent PPARγ SUMOylation [16] and that the PPARγ gene is not vital for the inhibition of iNOS by PPARγ agonists [25]. This implies that these candidates may have minor impact on PPARγ mediated gene expression but possible posttranscriptional regulation of PPARγ function.

Several plant derived natural products were shown to exert anti-inflammatory effects via inhibition of the NF-κB/iNOS pathway mediated NO production [33, 62–65]. Therefore, the screened candidate constituents may act by this mechanism and may contribute to the NO production inhibition effect by the extracts. Pharmacological modulation of iNOS activity has been achieved using structural analogs of arginine [66]. Agmatine (decarboxylated arginine), agmatine conjugates, and related guanidine derivatives (S1a Fig) were identified in *M. subcordata*. Reports support that aminoguanidine [48, 67] and agmatine [68–70] are selective iNOS inhibitors. Both compounds were shown to attenuate the activation of NF-κB and its pro-inflammatory target genes [67, 70] Therefore, agmatine and its conjugates could have partly contributed to the inhibition of LPS-induced NO production by *M. subcordata* extracts.

Besides, the inhibition by the other constituents such as stachydrine, trigonelline, the candidate isothiocyanates, and indole-3-carboxyaldyhyde could have contributed to the inhibition of NO production by *M. subcordata* extracts, possibly providing additive or synergistic effect as the activity of the extracts seems to be stronger than that of the individual candidate constituents that were tested. Indeed, adequate literature support exists on stachydrine, trigonelline, and the glucosinolate breakdown products. In vitro and in vivo studies show that stachydrine exhibits inhibition of the NF-κB signalling pathways [34, 71, 72]. Likewise, trigonelline showed antidiabetic effects and counteracted insulin resistance by suppressing inflammation [34, 73]. Also, ITCs have been linked with lower cancer risk [74, 75] via suppressing NF-κB signalling pathways that lead to attenuated pro-inflammatory mediators and activities. ITCs were shown to reduce several pro-inflammatory mediators and cytokines, including iNOS, apparently by downregulation of NF-κB signalling pathways [74, 76].

Taken together, the present study verified the induction of PPARγ mediated gene expression and the inhibition of LPS induced NO production by *M. subcordata* extracts and candidate constituents thereof. Different plant parts contain active compounds that act at multiple targets in the inflammatory response pathways and regulate a multitude of chemical mediators, enzymes, genes or cellular functions to alleviate inflammation [30] and hence, outcomes of assays that utilize extracts are expected to reflect a net effect of such potential multi-target interactions that may also apply to *M. subcordata* extracts. The PPARγ agonists’ ability to inhibit inflammatory responses by repressing NF-κB target genes has been linked to the prevention and treatment of the metabolic syndrome and diabetes [77, 78]. Likewise, the induction of PPARγ mediated gene expression as well as inhibition of NO production by *M. subcordata* extracts and candidate constituents thereof may imply that *M. subcordata* may have potential anti-inflammatory effects possibly by inhibiting NF-κB signalling pathways although the data in the present study are not enough to claim anti-inflammatory effects and that further studies are required. Results of the present study may also partly explain the use of the study plant in traditional medicine to manage infectious and chronic diseases associated to the metabolic syndrome.

## Conclusion

The present work showed for the first time the induction of PPARγ mediated gene expression by *M. subcordata* fruit, root, and seed extracts and the inhibition of LPS induced NO production in RAW264.7 macrophages by the fruit, leaf, root, and seed extracts and candidate constituents thereof that included agmatine, stachydrine, trigonelline, and glucosinolate breakdown products. Thus, additional in vivo studies on preparations or parts of this plant may be warranted so as to further verify its potential health benefits.

## Supplementary information

**Additional file 1: S1. Table.** Compounds tentatively identified as candidate constituents of *Maerua subcordata*. These compounds are reported in the literature to be ligands of PPARγ and/or influence PPARγ functions [47, 56, 61, 79–91].

**Additional file 2: S1. Fig**. Some candidate constituents tentatively identified in *M. subcordata* methanol extracts as displayed on MAGMa interface. (**a**) guanidine derivatives, (**b**) quaternary ammonium compounds (betaines), and (**c**) fatty acids and miscellaneous compounds.

**Additional file 3: S2. Fig.** Calibration curves. (**a**) standard calibration curve used to estimate LPS induced nitric oxide production, (**b**) standard calibration curves with and without addition of extracts to the standard nitrite in order to check for false negative/positive results due to possible intrinsic influence of the extracts on the absorbance at 540 nm, the wavelength used to measure levels of nitric oxide.

## Data Availability

All relevant data are included within the manuscript and its supporting information files. Yet, any additional raw data may be provided up on request if deemed necessary.

## Abbreviations

BSA: Bovine serum albumen
DCC-FCS: Dextran-coated charcoal-stripped foetal calf serum
DMEM/F12: Minimum Essential Medium alpha 1:1 mixture of Dulbecco’s modified Eagle’s medium and Ham’s F12 medium
DMSO: Dimethyl sulfoxide
FCS: Foetal calf serum
gDW/L: Gram dry weight per litre
iNOS: Inducible nitric-oxide synthase
LC-MS: Liquid chromatography (LC) coupled to multistage mass spectroscopy (MS)
LPS: Lipopolysaccharide
MAGMa: ‘Ms Annotation based on in silico Generated Metabolites’
MTT: 3-(4,5-dimethylthiazolyl)-2,5-diphenyltetrazolium bromide
NEAA: Nonessential amino acids
NED: N-1-napthylethylenediamine dihydrochloride
NF-κB: Nuclear factor kappaB
NO: Nitric oxide
PBS: Phosphate Buffer Saline
PPARs: Peroxisome proliferator activated receptors
RG: Rosiglitazone
SA: Sulfanilamide

## References

[1] Lee J, Jo DG, Park D, Chung HY, Mattson MP (2014). Adaptive cellular response pathways adaptive cellular stress pathways as therapeutic targets of dietary phytochemicals: focus on the nervous system. Pharmacol Rev.

[2] Howes MJ, Simmonds MS (2014). The role of phytochemicals as micronutrients in health and disease. Curr Opin Clin Nutr Metab Care.

[3] Upadhyay S, Dixit M (2015). Role of polyphenols and other phytochemicals on molecular signaling. Oxid Med Cellular Longevity.

[4] Ko JK, Lee SS, Martin H (2010). Phytochemicals as Modulators of PPARs and RXRs. PPAR Res.

[5] Kota BP, Huang THW, Roufogalis BD (2005). An overview on biological mechanisms of PPARs. Pharmacol Res.

[6] Corzo C, Griffin PR (2013). Targeting the peroxisome proliferator-activated receptor-γ to counter the inflammatory milieu in obesity. Diabetes Metab J.

[7] Han L, Shen WJ, Bittner S, Kraemer FB, Azhar S (2017). PPARs: regulators of metabolism and as therapeutic targets in cardiovascular disease. Part II: PPAR-β/δ and PPAR-γ. Futur Cardiol.

[8] Botta Margherita, Audano Matteo, Sahebkar Amirhossein, Sirtori Cesare, Mitro Nico, Ruscica Massimiliano (2018). PPAR Agonists and Metabolic Syndrome: An Established Role?. International Journal of Molecular Sciences.

[9] Liu T, Zhang L, Joo D, Sun SC. NF-κB signaling in inflammation. Signal Transduct Target Ther. 2017;2.pii:17023. 10.1038/sigtrans.2017.23 PMC5661633 29158945

[10] Wahli W, Michalik L (2012). PPARs at the crossroads of lipid signaling and inflammation. Trends Endocrinol Metabol.

[11] Picard F, Auwerx J (2002). PPAR (gamma) and glucose homeostasis. Annu Rev Nutr.

[12] Gijsbers L, Man HY, Kloet SK, de Haan LHJ, Keijer J, Rietjens IMCM (2011). Stable reporter cell lines for peroxisome proliferator-activated receptor γ (PPARγ)-mediated modulation of gene expression. Anal Biochem.

[13] Brunmeir Reinhard, Xu Feng (2018). Functional Regulation of PPARs through Post-Translational Modifications. International Journal of Molecular Sciences.

[14] Varga T, Czimmerer Z, Nagy L (2011). PPARs are a unique set of fatty acid regulated transcription factors controlling both lipid metabolism and inflammation. Biochim Biophys Acta.

[15] Mirza AZ, Althagafi II, Shamshad H (2019). Role of PPAR receptor in different diseases and their ligands: physiological importance and clinical implications. Eur J Med Chem.

[16] Pascual G, Fong AL, Ogawa S, Gamliel A, Li AC, Perissi V (2005). A SUMOylation-dependent pathway mediates transrepression of inflammatory response genes by PPAR-gamma. Nature.

[17] Zelcer N, Tontonoz P (2005). SUMOylation and PPARgamma: wrestling with inflammatory signaling. Cell Metab.

[18] Baker RG, Hayden MS, Ghosh S (2011). NF-kB, inflammation, and metabolic disease. Cell Metabol.

[19] Napetschnig J, Wu H (2013). Molecular basis of NF-κB signaling. Annu Rev Biophys.

[20] Minihane AM, Vinoy S, Russell WR, Baka A, Roche HM, Tuohy KM (2015). Low-grade inflammation, diet composition and health: current research evidence and its translation. Br J Nutr.

[21] Aktan F (2004). iNOS-mediated nitric oxide production and its regulation. Life Sci.

[22] Choudhury MG, Kumari S, Das KB, Saha N (2018). Lipopolysaccharide causes NFĸB-mediated induction of inducible nitric oxide synthase gene and more production of nitric oxide in air-breathing catfish, Clarias Magur (Hamilton). Gene.

[23] Wen Q, Mei L, Ye S, Liu X, Xu Q, Miao J (2018). Chrysophanol demonstrates anti-inflammatory properties in LPS-primed RAW 264.7 macrophages through activating PPAR-γ. Int Immunopharmacol.

[24] Lin CF, Young KC, Bai CH, Yu BC, Ma CT, Chien YC (2014). Rosiglitazone Regulates Anti-Inflammation and Growth Inhibition via PTEN. Biomed Res Int.

[25] Crosby MB, Zhang J, Nowling TM, Svenson JL, Nicol CJ, Gonzalez FJ (2006). Inflammatory modulation of PPARγ expression and activity. Clin Immunol.

[26] Kim J, Park CS, Lim Y, Kim HS (2009). Paeonia japonica, Houttuynia cordata, and Aster scaber water extracts induce nitric oxide and cytokine production by lipopolysaccharide-activated macrophages. J Med Food.

[27] Hiben MG, Louisse J, de Haan LHJ, Rietjens IMCM. Ethnomedicine and ethnobotany of *Maerua subcordata* (Gilg) DeWolf. J Ethnic Foods 2019a; 6:23. https://doi.org/10.1186/s42779-019-0032-4..

[28] Calder PC, Ahluwalia N, Brouns F, Buetler T, Clement K, Cunningham K (2011). Dietary factors and low-grade inflammation in relation to overweight and obesity. Br J Nutr.

[29] Stefanson AL, Bakovic M (2014). Dietary regulation of Keap1/Nrf2/ARE pathway: focus on plant-derived compounds and trace minerals. Nutrients.

[30] Dar KB, Bhat AH, Amin S, Masood A, Zargar MA, Ganie SA (2016). Inflammation: a multidimensional insight on natural anti-inflammatory therapeutic compounds. Curr Med Chem.

[31] Mazumder A, Dwivedi A, du Plessis J (2016). Sinigrin and its therapeutic benefits. Molecules.

[32] Ndoye Foe FM, Tchinang TFK, Nyegue AM, Abdou JP, Yaya AJG, Tchinda AT (2016). Chemical composition, in vitro antioxidant and anti-inflammatory properties of essential oils of four dietary and medicinal plants from Cameroon. BMC Complement Altern Med.

[33] Subedi L, Venkatesan R, Kim SY. Neuroprotective and Anti-Inflammatory Activities of Allyl Isothiocyanate through Attenuation of JNK/NF-κB/TNF-α Signaling. Int J Mol Sci. 2017;18(7). pii: E1423. 10.3390/ijms18071423 PMC5535914 28671636

[34] Liu Y, Wei S, Zou Q, Luo Y (2018). Stachydrine suppresses viability & migration of astrocytoma cells via CXCR4/ERK & CXCR4/Akt pathway activity. Future Oncol.

[35] Gebrelibanos Hiben M, de Haan L, Spenkelink B, Wesseling S, Louisse J, Vervoort J (2019). Effects of *Maerua subcordata* (Gilg) DeWolf on electrophile-responsive element (EpRE)-mediated gene expression *in vitro*. PLoS One.

[36] Gijsbers L, van Eekelen HDLM, Nguyen TH, de Haan LHJ, van der Burg B, Aarts JMMJG (2012). Induction of electrophile-responsive element (EpRE)-mediated gene expression by tomato extracts in vitro. Food Chem.

[37] van der Linden SC, von Bergh ARM, van Vught-Lussenburg BMA, Jonker LRA, Teunis M, Krul CAM (2014). Development of a panel of high-throughput reporter-gene assays to detect genotoxicity and oxidative stress. Mutat Res Genet Toxicol Environ Mutagen.

[38] Gijsbers L, van Eekelen HDLM, de Haan LHJ, Swier JM, Heijink NL, Kloet SK (2013). Induction of Peroxisome Proliferator-Activated Receptor γ (PPARγ)- Mediated Gene Expression by Tomato (*Solanum lycopersicum* L.) Extracts. J Agr Food Chem.

[39] Beekmann K, Rubió L, de Haan LH, Actis-Goretta L, van der Burg B, van Bladeren PJ (2015). The effect of quercetin and kaempferol aglycones and glucuronides on peroxisome proliferator-activated receptor-gamma (PPAR-γ). Food Funct.

[40] Bowdish D Propagation & Culturing of RAW264.7 Cells. Bowdish Lab, McMaster University (www.bowdish.ca); Hamilton, ON, Canada; 2013.

[41] Meijerink J, Plastina P, Vincken JP, Poland M, Attya M, Balvers M (2011). The ethanolamide metabolite of DHA, docosahexaenoylethanolamine, shows immunomodulating effects in mouse peritoneal and RAW264.7 macrophages: evidence for a new link between fish oil and inflammation. Br J Nutr.

[42] Sun J, Zhang X, Broderick M, Fein H (2003). Measurement of nitric oxide production in biological systems by using Griess reaction assay. Sensors.

[43] Králová J, Pekarová M, Drábiková K, Jančinová V, Nosáľ R, Číž M (2008). The effects of dithiaden on nitric oxide production by RAW 264.7 cells. Interdisc Toxicol.

[44] Promega. Griess Reagent System Technical Bulletin; 2800 Woods Hollow Road Madison, USA;2009.

[45] Ridder L, van der Hooft JJJ, Verhoeven S, de Vos RCH, van Schaik R, Vervoort J (2012). Substructure-based annotation of high-resolution multistage MS^n^ spectral trees. Rapid Commun Mass Spectrom.

[46] Queiroz TB, Santos GF, Ventura SC, Hiruma-Lima CA, Gaivão IOM, Maistro EL (2017). Cytotoxic and genotoxic potential of geraniol in peripheral blood mononuclear cells and human hepatoma cell line (HepG2). Genet Mol Res.

[47] Nissim I, Horyn O, Daikhin Y, Chen P, Li C, Wehrli SL (2014). The molecular and metabolic influence of long term agmatine consumption. J Biol Chem.

[48] Misko TP, Moore WM, Kasten TP, Nickols GA, Corbett JA, Tilton RG (1993). Selective inhibition of the inducible nitric oxide synthase by aminoguanidine. Eur J Pharmacol.

[49] Assreuy J, Cunha FQ, Epperlein M, Noronha-Dutra A, O'Donnell CA, Liew FY (1994). Production of nitric oxide and superoxide by activated macrophages and killing of Leishmania major. Eur J Immunol.

[50] Lupinacci E, Meijerink J, Vincken JP, Gabriele B, Gruppen H, Witkamp RF (2009). Xanthohumol from hop (*Humulus lupulus* L.) is an efficient inhibitor of monocyte Chemoattractant Protein-1 and tumor necrosis factor-α release in LPS-stimulated RAW 264.7 mouse macrophages and U937 human monocytes. J Agric Food Chem.

[51] Choi EM, Hwang JK (2005). Screening of Indonesian medicinal plants for inhibitor activity on nitric oxide production of RAW264.7 cells and antioxidant activity. Fitoterapia.

[52] Conforti F, Menichini F (2011). Phenolic compounds from plants as nitric oxide production inhibitors. Curr Med Chem.

[53] Xu HE, Lambert MH, Montana VG, Parks DJ, Blanchard SG, Brown PJ (1999). Molecular recognition of fatty acids by peroxisome proliferator–activated receptors. Mol Cell.

[54] Schupp M, Lazar MA (2010). Endogenous ligands for nuclear receptors: digging deeper. J Biol Chem.

[55] Yun C, DasGupta R (2014). Luciferase reporter assay in *Drosophila* and mammalian tissue culture cells. Curr Protoc Chem Biol.

[56] Kendrick N. A gene’s mRNA level does not usually predict its protein level. Kendrick Labs, Inc.;2014.

[57] Jeyam M, Priyadharsini K, Ravikumar P, Shalini G (2014). Evaluating multi-target efficiency of phytocompounds against diabetes mellitus - an *in silico* approach. BIOINFO Drug Targets.

[58] Kuchta K, Matsuura N, Rauwald HW, Iinuma M. Effects of *Leonurus japonicus* Houtt. and its N-containing constituents leonurine and stachydrine on the activity of PPARα, β/δ, and γ in a newly developed in vitro luciferase reporter gene assay. Planta Med. 2014;80-P1L24.

[59] Tharaheswari M, Jayachandra Reddy N, Kumar R, Varshney KC, Kannan M, Sudha RS (2014). Trigonelline and diosgenin attenuate ER stress, oxidative stress-mediated damage in pancreas and enhance adipose tissue PPARγ activity in type 2 diabetic rats. Mol Cell Biochem.

[60] Aoyagi R, Funakoshi-Tago M, Fujiwara Y, Tamura H (2014). Coffee inhibits adipocyte differentiation via inactivation of PPARγ. Biol Pharm Bull.

[61] Ilavenil S, Kim DH, Jeong YI, Arasu MV, Vijayakumar M, Prabhu PN (2015). Trigonelline protects the cardiocyte from hydrogen peroxide induced apoptosis in H9c2 cells. Asian Pacific J Trop Med.

[62] Senchina DS, Martin AE, Buss JE, Kohut ML (2010). Effects of Echinacea extracts on macrophage antiviral activities. Phytother Res.

[63] Ding HY, Wu PS, Wu MJ. *Cleome rutidosperma* and *Euphorbia thymifolia* Suppress Inflammatory Response via Upregulation of Phase II Enzymes and Modulation of NF-κB and JNK Activation in LPS-Stimulated BV2 Microglia. Int J Mol Sci. 2016;17(9):pii: E1420. 10.3390/ijms17091420 PMC5037699 27618898

[64] Xu J, Zhao Y, Aisa HA (2017). Anti-inflammatory effect of pomegranate flower in lipopolysaccharide (LPS)-stimulated RAW264.7 macrophages. Pharm Biol.

[65] Sun Weixiang, Gao Yuyan, Ding Yushi, Cao Ying, Chen Jing, Lv Gaohong, Lu Jinfu, Yu Bin, Peng Meilin, Xu Huiqin, Sun Yun (2019). Catalpol ameliorates advanced glycation end product‐induced dysfunction of glomerular endothelial cells via regulating nitric oxide synthesis by inducible nitric oxide synthase and endothelial nitric oxide synthase. IUBMB Life.

[66] Billack B, Heck DE, Porterfield DM, Malchow RP, Smith PJ, Gardner CR (2001). Minimal amidine structure for inhibition of nitric oxide biosynthesis. Biochem Pharmacol.

[67] Natarajan K, Abraham P, Kota R, Isaac B (2018). NF-κB-iNOS-COX2-TNF α inflammatory signaling pathway plays an important role in methotrexate induced small intestinal injury in rats. Food Chem Toxicol.

[68] Auguet M, Viossat I, Marin JG, Chabrier PE (1995). Selective inhibition of inducible nitric oxide synthase by agmatine. Jpn J Pharmacol.

[69] Regunathan S, Piletz JE (2003). Regulation of inducible nitric oxide synthase and agmatine synthesis in macrophages and astrocytes. Ann N Y Acad Sci.

[70] Ahmed N, Aljuhani N, Al-Hujaili HS, Al-Hujaili MA, Elkablawy MA, Noah MM (2018). Agmatine protects against sodium valproate-induced hepatic injury in mice via modulation of nuclear factor-κB/inducible nitric oxide synthetase pathway. J Biochem Mol Toxicol.

[71] Chen HH, Zhao P, Zhao WX, Tian J, Guo W, Xu M (2017). Stachydrine ameliorates pressure overload-induced diastolic heart failure by suppressing myocardial fibrosis. Am J Transl Res.

[72] Zhang J, Yang A, Wu Y, Guan W, Xiong B, Peng X (2018). Stachydrine ameliorates carbon tetrachloride-induced hepatic fibrosis by inhibiting inflammation, oxidative stress and regulating MMPs/TIMPs system in rats. Biomed Pharmacother.

[73] Khound Rituraj, Shen Jing, Song Yongyan, Santra Dipak, Su Qiaozhu (2018). Phytoceuticals in Fenugreek Ameliorate VLDL Overproduction and Insulin Resistance via the Insig Signaling Pathway. Molecular Nutrition & Food Research.

[74] Prawan A, Saw CL, Khor TO, Keum YS, Yu S, Hu L (2009). Anti-NF-kappaB and anti-inflammatory activities of synthetic isothiocyanates: effect of chemical structures and cellular signaling. Chem Biol Interact.

[75] Wu X, Zhou Q, Xu K (2009). Are isothiocyanates potential anti-cancer drugs?. Acta Pharmacol Sin.

[76] Cho HJ, Lee KW, Park JH (2013). Erucin exerts anti-inflammatory properties in murine macrophages and mouse skin: possible mediation through the inhibition of NFκB signaling. Int J Mol Sci.

[77] Hirai S, Takahashi N, Goto T, Lin S, Uemura T, Yu R (2010). Functional food targeting the regulation of obesity-induced inflammatory responses and pathologies. Mediators Inflamm.

[78] Zhou JY, Du XH, Zhang Z, Qian GS (2017). Trigonelline inhibits inflammation and protects β cells to prevent fetal growth restriction during pregnancy in a mouse model of diabetes. Pharmacol.

[79] Cobb JE, Blanchard SG, Boswell EG, Brown KK, Charifson PS, Cooper JP (1998). N-(2-Benzoylphenyl)-L-tyrosine PPARgamma agonists. 3. Structure-activity relationship and optimization of the N-aryl substituent. J Med Chem.

[80] Merk D, Lamers C, Weber J, Flesch D, Gabler M, Proschak E (2015). Anthranilic acid derivatives as nuclear receptor modulators-development of novel PPAR selective and dual PPAR/FXR ligands. Bioorg Med Chem.

[81] Prabhakar PK, Doble M (2011). Effect of natural products on commercial Oral Antidiabetic drugs in enhancing 2-Deoxyglucose uptake by 3T3-L1 adipocytes. Ther Adv Endocrinol Metab.

[82] Mastrofrancesco A, Ottaviani M, Aspite N, Cardinali G, Izzo E, Graupe K (2010). Azelaic acid modulates the inflammatory response in normal human keratinocytes through PPARgamma activation. Exp Dermatol.

[83] Chang HP, Wang ML, Hsu CY, Liu ME, Chan MH, Chen YH (2011). Suppression of inflammation-associated factors by indole-3-carbinol in mice fed high-fat diets and in isolated, co-cultured macrophages and adipocytes. Int J Obes.

[84] Patel B, Mann GE, Chapple SJ (2018). Concerted redox modulation by sulforaphane alleviates diabetes and cardiometabolic syndrome. Free Rad Biol Med.

[85] Nguyen MT, Csermely P, Soti C (2013). Hsp90 chaperones PPARc and regulates differentiation and survival of 3T3-L1 adipocytes. Cell Death Differ.

[86] Schopfer FJ, Cole MP, Groeger AL, Chen CS, Khoo NKH, Woodcock SR (2010). Covalent peroxisome proliferator-activated receptor γ adduction by nitro-fatty acids. J Biol Chem.

[87] Wang KC, Tsai CP, Lee CL, Chen SY, Lin GJ, Yen MH (2013). α-Lipoic acid enhances endogenous peroxisome-proliferator-activated receptor-γ to ameliorate experimental autoimmune encephalomyelitis in mice. Clin Sci (Lond).

[88] Edwards IJ, OFlaherty JT (2008). Omega-3 fatty acids and PPAR*γ* in Cancer. PPAR Res.

[89] Fujimori K (2012). Prostaglandins as PPAR*γ* modulators in Adipogenesis. PPAR Res.

[90] Jeong HW, Lee JW, Kim WS, Choe SS, Shin HJ, Lee GY (2010). A nonthiazolidinedione peroxisome proliferator-activated receptor α/γ dual agonist CG301360 alleviates insulin resistance and lipid Dysregulation in *db/db* mice. Mol Pharmacol.

[91] Nencu I, Vlase L, Istudor V, Mircea T (2015). Preliminary research regarding *Urtica urens* l. and *Urtica dioica* L. Farmacia.

