# Supplementary material for: Induction of peroxisome proliferator activated receptor γ (PPARγ) mediated gene expression and inhibition of induced nitric oxide production by Maerua subcordata (Gilg) DeWolf
Source: BMC Complement Med Ther. 2020 Mar 12;20:80. doi: 10.1186/s12906-020-2856-2 (PMC7076844; doi:10.1186/s12906-020-2856-2)
Supplement: Supplementary file 1 — Additional file 1: S1. Table. Compounds tentatively identified as candidate constituents of Maerua subcordata. These compounds are reported in the literature to be ligands of PPARγ and/or influence PPARγ functions [47, 56, 61, 79–91]. [file 12906_2020_2856_MOESM1_ESM.docx]

**S1 Table.** Compounds tentatively identified as candidate constituents of *Maerua subcordata*. These compounds are reported in the literature to be ligands of PPARγ and/or influence PPARγ functions [47, 56, 61, 79-91].

| Compound | Literature |
| --- | --- |
| Agmatine | Agmatine increased gene expression of PPARα and PPARγ *in vivo* in rats [47]. |
| Anthranilic acid | Anthranilic acid derivatives were reported as novel selective PPAR ligands [79,80]. |
| Arecaidine | Arecaidine derivative, arecaidine methyl ester (arecoline) increased the translocation of glucose transporter type 4 *via* the PPARγ pathway [81]. |
| Azelaic acid | Azelaic acid induced PPARγ mRNA and its transcriptional activity in human keratinocytes [82]. |
| Indole-3-carboxaldehyde  (derivative of indole-3-carbinol) | Indole-3-carbinol decreased expression of iNOS, decreased nitrite content and enhanced expression of PPARγ *in vitro* [83]. |
| Isothiocyanates | Sulforaphane modulates NFκB and PPARγ signalling [84]. |
| Geranylgeranylacetone (Teprenone) | Possible boosting of optimal PPARγ was suggested mechanism by which geranylgeranylacetone promote weight loss or improve insulin resistance in rodents and humans [85]. |
| 9-HODE | 9-Hydroxyoctadecadienoic acid (9-HODE) is among the most proposed natural PPAR γ ligands [86]. |
| α-Lipoic acid | α-Lipoic acid acts as a PPARγ agonist to counteract oxidative stress [87]. |
| *α*-Linolenic acid | Polyunsaturated fatty acids including *α*-linolenic acid are natural ligands for PPARγ [88]. |
| Petroselinic acid | Fatty acids including petroselinic acid and linolenic acid were identified as PPAR*γ* ligands [89]. |
| Pipecolic acid  (piperidine-2-carboxylic acid) | A pipecolic acid derivative stimulated transcriptional activities of PPARα and PPARγ and induced expression of their target genes [90]. |
| Stachydrine | Stachydrine showed a PPARγ receptor glide score comparable to synthetic antidiabetic drugs [56]. |
| Stigmasterol | Sterols like stigmasterol are known PPARγ agonists (can lower blood glucose) [91]. |
| Trigonelline | Trigonelline increased insulin sensitivity and enhanced adipose tissue PPARγ activity in diabetic rats *in vivo* [61]. |
