# Supplementary material for: Induction of peroxisome proliferator activated receptor γ (PPARγ) mediated gene expression and inhibition of induced nitric oxide production by Maerua subcordata (Gilg) DeWolf
Source: BMC Complement Med Ther. 2020 Mar 12;20:80. doi: 10.1186/s12906-020-2856-2 (PMC7076844; doi:10.1186/s12906-020-2856-2)
Supplement: Supplementary file 2 — Additional file 2: S1. Fig. Some candidate constituents tentatively identified in M. subcordata methanol extracts as displayed on MAGMa interface. (a) guanidine derivatives, (b) quaternary ammonium compounds (betaines), and (c) fatty acids and miscellaneous compounds. [file 12906_2020_2856_MOESM2_ESM.pdf]

(S1a Fig) Guanidine derivatives

Home Help Workspace
Logout Information

MAGMa

netherlands eScience cente

Molecules

Scans	Assigned	Candidate score	Molecule	Formula	Mass	ΔMass (ppm)	Name	Reactions
2	No	3.65816	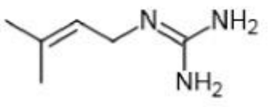	C6H13N3	127.11100	0.19482	Galegine (10983)	
3	No	0.78767	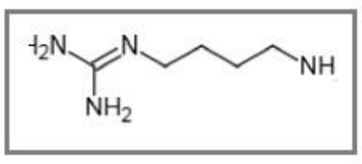	C5H14N4	130.12200	-1.65939	agmatine (199)	
1	No	4.03146	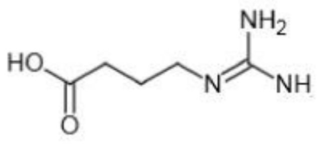	C5H11N3O2	145.08500	-0.17917	4-Guanidinobutyric acid (500)	
1	No	5.74754	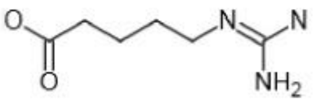	C6H13N3O2	159.10100	-3.05011	delta-Guanidinovaleric acid (160464)	
2	No	3.67827	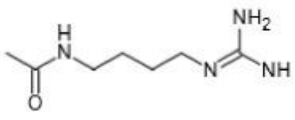	C7H16N4O	172.13200	0.20973	Acetylagmatine (439661)	
1	No	4.99708	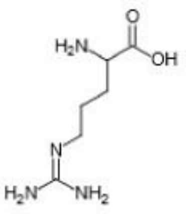	C6H14N4O2	174.11200	-2.04184	arginine (232)	
3	No	3.83924	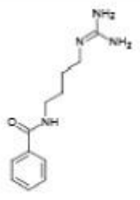	C12H18N4O	234.14800	0.03536	Benzoylagmatine (439689)	
10	No	2.29063	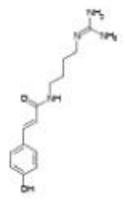	C14H20N4O2	276.15900	-0.51883	p-Coumaroylagmatine (5280691)	
19	No	2.33180	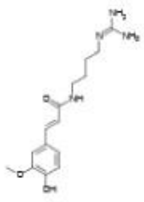	C15H22N4O3	306.16900	1.06426	feruloylagmatine (46173376)	

(S1b Fig) Quaternary ammonium compounds (betaines)

Home Help Workspace
Logout Information

MAGMa

netherlands eScience center

Molecules

Scans	Assigned	Candidate score	Molecule	Formula	Mass	ΔMass (ppm)	Name	Reactions
2	No	4.09491	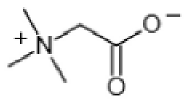	C5H11NO2	117.07900	1.58779	betaine (247)	
3	No	0.93510	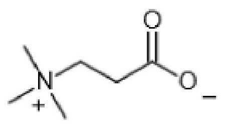	C6H13NO2	131.09500	-2.74773	Propiobetaine (441440)	
1	No	1.04094	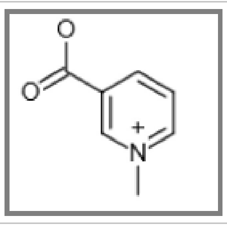	C7H7NO2	137.04800	-2.82908	Trigonelline (5570)	
27	No	4.89969	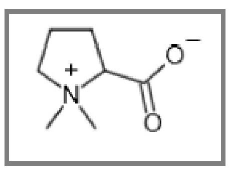	C7H13NO2	143.09500	-3.04836	stachydrine (115244)	
1	No	3.13020	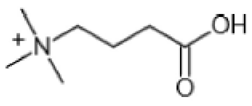	C7H16NO2+	146.11800	-0.03080	gamma-butyrobetaine (134)	
1	No	3.86928	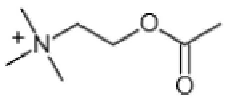	C7H16NO2+	146.11800	-0.03080	acetylcholine (187)	
3	No	8.25592	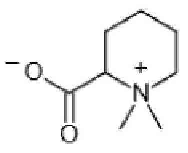	C8H15NO2	157.11000	1.85013	Homostachydrine (4479243)	
22	No	1.97166	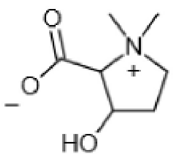	C7H13NO3	159.08954	-0.77757	3-Hydroxystachydrine (46173781)	

(S1c Fig) Fatty acids and miscellaneous compounds

Home Help Workspace
Logout Information

MAGMa

netherlands eScience center

Molecules

Scans	Assigned	Candidate score	Molecule	Formula	Mass	ΔMass (ppm)	Name	Reactions
1	No	1.90721	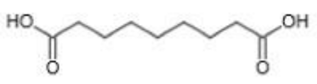	$C_9H_{16}O_4$	188.10500	-1.58313	azelaic acid (2266)	
5	No	3.20466	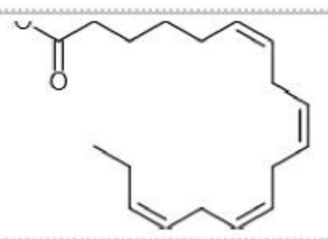	$C_{18}H_{28}O_2$	276.20900	-2.85631	Stearidonic acid (5312508)	
6	No	4.93568	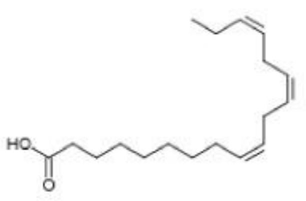	$C_{18}H_{30}O_2$	278.22500	-4.06937	alpha-Linolenic acid (5280934)	
1	No	4.74950	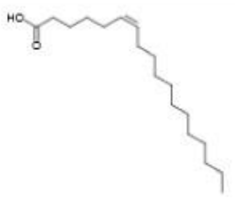	$C_{18}H_{34}O_2$	282.25600	-2.26699	PETROSELINIC ACID (5281125)	
8	No	1.96226	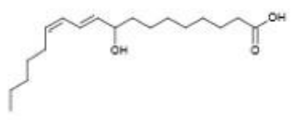	$C_{18}H_{32}O_3$	296.23500	0.30537	9(S)-HODE (5312830)	
8	No	1.49024	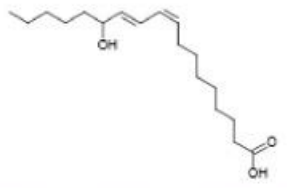	$C_{18}H_{32}O_3$	296.23500	0.09863	13(S)-HODE (6443013)	
9	No	3.52517	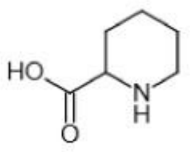	$C_6H_{11}NO_2$	129.07900	0.20970	Pipecolic acid (439227)	
1	No	2.54728	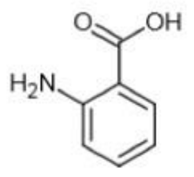	$C_7H_7NO_2$	137.04768	-1.38089	anthranilic acid (227)	
2	No	7.39997	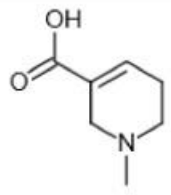	$C_7H_{11}NO_2$	141.07900	-1.63366	Arecaidine (10355)	
18	No	4.46319	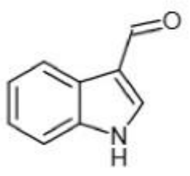	C_9H_7NO	145.05276	-0.27682	INDOLE-3-CARBOXALDEHYDE (10256)	
22	No	5.24249	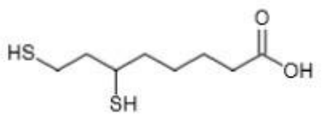	$C_8H_{16}O_2S_2$	208.05900	-3.96157	Dihydrolipoic acid (421)	
1	No	5.78280	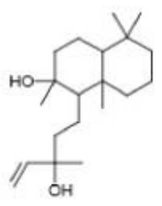	$C_{20}H_{36}O_2$	308.27200	-3.88084	Sclareol (163263)	
1	No	0.32167	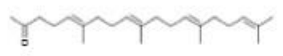	$C_{23}H_{38}O$	330.29200	-0.15569	teprenone (5282199)	
1	No	3.85601	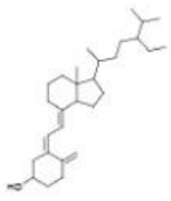	$C_{29}H_{48}O$	412.37100	-3.12739	Vitamin D5 (9909623)	
1	No	3.88799	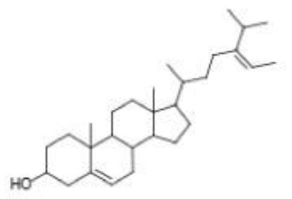	$C_{29}H_{48}O$	412.37100	-3.12739	Isofucosterol (5281326)	
1	No	3.92162	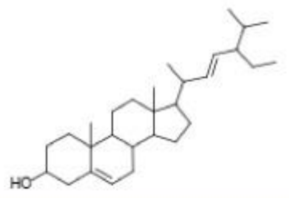	$C_{29}H_{48}O$	412.37100	-3.12739	STIGMASTEROL (5280794)	
