# Supplementary figures and images for: Induction of peroxisome proliferator activated receptor γ (PPARγ) mediated gene expression and inhibition of induced nitric oxide production by Maerua subcordata (Gilg) DeWolf
Source: BMC Complement Med Ther. 2020 Mar 12;20:80. doi: 10.1186/s12906-020-2856-2 (PMC7076844; doi:10.1186/s12906-020-2856-2)

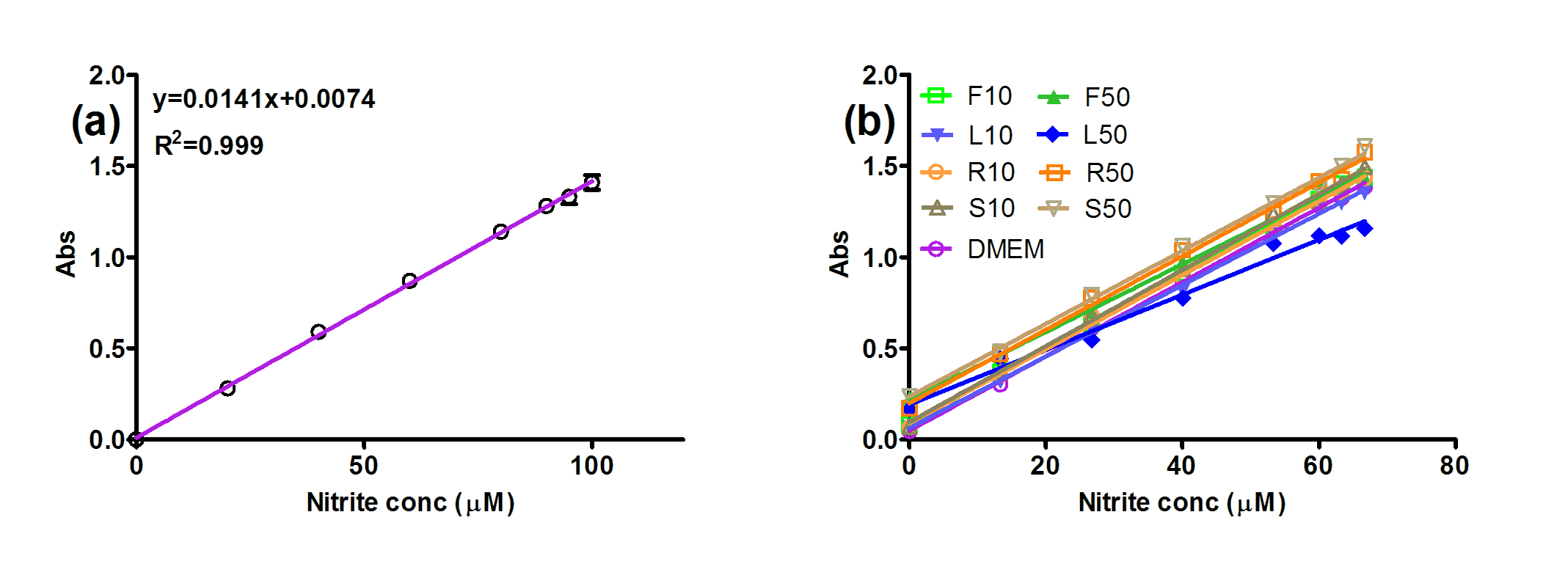

Supplement: Supplementary file 3 — Additional file 3: S2. Fig. Calibration curves. (a) standard calibration curve used to estimate LPS induced nitric oxide production, (b) standard calibration curves with and without addition of extracts to the standard nitrite in order to check for false negative/positive results due to possible intrinsic influence of the extracts on the absorbance at 540 nm, the wavelength used to measure levels of nitric oxide. [file 12906_2020_2856_MOESM3_ESM.tif]
